# Association Between Serum Galectin‐3 and Parkinson's Disease: A Two‐Sample Mendelian Randomization Study

**DOI:** 10.1002/brb3.70103

**Published:** 2024-10-23

**Authors:** Rui Pan, Wei Li, Jinyuan Wang, Jiarong Xie, Xiucan Weng, Ying Yang, Xiaolei Shi

**Affiliations:** ^1^ School of Nursing Huizhou Health Sciences Polytechnic Huizhou Guangdong Province P. R. China; ^2^ School of Clinical Medicine Huizhou Health Sciences Polytechnic Huizhou Guangdong Province P. R. China; ^3^ Department of Neurology Sun Yat‐Sen Memorial Hospital, Sun Yat‐Sen University Guangzhou Guangdong Province P. R. China; ^4^ Department of Neurology The Affiliated Brain Hospital of Guangzhou Medical University Guangzhou Guangdong Province P. R. China; ^5^ School of Mental Health Guangzhou Medical University Guangzhou Guangdong Province P. R. China; ^6^ Institute of Psychiatry and Psychology Guangzhou Medical University Guangzhou Guangdong Province P. R. China; ^7^ Key Laboratory of Neurogenetics and Channelopathies of Guangdong Province and the Ministry of Education of China Guangzhou Medical University Guangzhou Guangdong Province P. R. China; ^8^ Guangdong Engineering Technology Research Center for Translational Medicine of Mental Disorders Guangzhou Medical University Guangzhou Guangdong Province P. R. China

**Keywords:** galectin‐3, genetic instrument, Mendelian randomization, Parkinson's disease

## Abstract

**Background:**

Parkinson's disease (PD) is a prevalent neurodegenerative disorder with poor prognosis. Observational studies have demonstrated a significant correlation between serum galectin‐3 and PD, suggesting a potential role of galectin‐3 as a biomarker for PD. However, it is still unclear whether galectin‐3 contributes to the risk of the disease.

**Methods:**

A two‐sample Mendelian randomization (MR) approach was used in this study. Genetic instruments for serum galectin‐3 level were selected from a genome‐wide association study (GWAS), including 30,931 European individuals. Summary‐level statistics for PD were derived from another published GWAS, including 33,674 cases and 449,056 controls. Primary analysis was conducted using the inverse‐variance weighting (IVW) method. Weighted median, MR‐Egger, simple mode, weighted mode, and MR‐pleiotropy residual sum and outlier (MR‐PRESSO) methods were used as complementary analyses. To detect heterogeneity, Cochran's *Q* statistic and leave‐one‐out analysis were used. For testing potential horizontal pleiotropy, the MR‐Egger intercept test and MR‐PRESSO global test were conducted.

**Results:**

MR analysis using IVW model (OR 1.112, 95% CI 1.025–1.206, *p* = 0.010), weighted median (OR 1.135, 95% CI 1.037–1.242, *p* = 0.006), weighted mode (OR 1.142, 95% CI 1.038–1.257, *p* = 0.030), and MR‐PRESSO (OR 1.112, 95% CI 1.046–1.182, *p* = 0.012) presented a consistent result, indicating that increased serum galectin‐3 was associated with a higher risk of PD. No heterogeneity or horizontal pleiotropy was detected in the analyses.

**Conclusions:**

The study shows a suggestive association between galectin‐3 and PD. Increasing serum galectin‐3 was associated with an increase in PD risk. Galectin‐3 may play an important role in the causal pathway to PD.

## Graphical abstract

A suggestive association between genetic susceptibility to serum galectin‐3 and risk of Parkinson's disease (PD) was demonstrated. Galectin‐3 may play an important role in the causal pathway to PD.

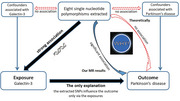

## 1 Introduction

Parkinson's disease (PD) is a chronic neurodegenerative disorder characterized by tremor, motor symptoms (rigidity, akinesia, etc.), and cognitive impairments (Bloem, Okun, and Klein 2021). The pathological hallmarks of the disease involve the accumulation of cytoplasmic inclusions called Lewy bodies (LBs) and the selective loss of dopaminergic neurons in the substantia nigra (SN). While many environmental and genetic factors have been reported to be involved in the onset and progression of PD, the exact etiology of the disorder remains poorly understood. Accumulating evidence has indicated that the interplay of the aberrant *α*‐synuclein aggregation, commonly known as the main component of LBs, and neuroinflammation in the SN significantly contribute to PD (Spillantini et al. 1997; Gelders, Baekelandt, and Perren 2018; Surguchov 2015). Therefore, growing attention has been paid to the investigation of novel biomarkers for the disorder.

Galectin‐3 is a multifunctional molecule that belongs to the carbohydrate‐ligand lectin family. It is widely expressed in the cytoplasm as well as the extracellular area of human tissues (Hara et al. 2020). In recent years, studies have suggested that galectin‐3 plays an important role in neuroinflammation and neurodegeneration (Boza‐Serrano et al. 2018; Ashraf and Baeesa 2018). It is regarded as a promoter of immune cell recruitment and participates in different stages of inflammation by activating lymphocytes, macrophages, and microglia (Tiwari and Pal 2017). Moreover, increased galectin‐3 can be measured in the serum of PD patients (Yazar, Yazar, and Cihan 2019; Cengiz et al. 2019). It has been used to aid in the early diagnosis and prognosis of the disease (Wu et al. 2021). Additionally, some researchers have revealed the noninflammatory aspects of galectin‐3. Burbidge et al. (2022) reported the presence of galectin‐3 in *α*‐synuclein deposits from LBs in post‐mortem brain samples of PD patients. Dilsizoglu et al. (2021) described the recruitment of galectin‐3 to damaged lysosomes bearing *α*‐synuclein fibrils in catecholaminergic neuronal cells. However, the above evidence may only indicate the association between galectin‐3 and PD. It is still doubtful whether there are causative effects of galectin‐3 on the risk of PD.

Mendelian randomization (MR) is an epidemiological method that uses genetic variants to explore the causality between exposure and outcome (Lawlor et al. 2008; Verduijn et al. 2010). The method typically employs single nucleotide polymorphisms (SNPs) as proxies for the exposure factor. As a variation at a single nucleotide site in the genome, SNP is randomly assigned at conception and will not be affected by environmental or lifestyle factors. Therefore, MR analysis can effectively eliminate confounding factors and provide an unbiased causal effect. In this study, we used a two‐sample MR method to investigate the genetic validity of serum galectin‐3 in the risk of PD.

## 2 Materials and Methods

### 2.1 Study Design

**FIGURE 1:**
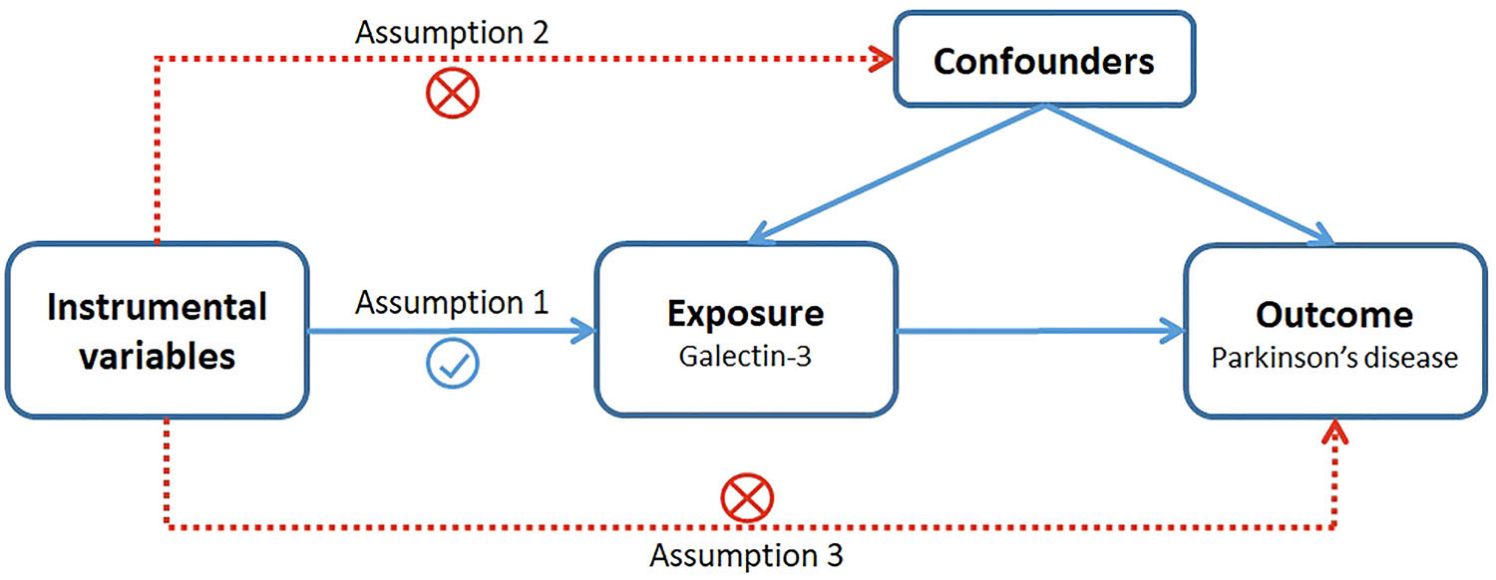
An overview of Mendelian randomization (MR) analysis in the study. MR is based on three assumptions. Assumption 1, SNPs identified as instrumental variables are supposed to be strongly correlated with exposure. Assumption 2, selected SNPs should be independent of any confounding factors. Assumption 3, the genetic variants are associated with the risk of outcome only via exposure, rather than through a direct association.

A univariable two‐sample MR method was used to investigate the relationship between galectin‐3 levels and PD. The workflow of our MR framework is shown in Figure 1. Generally, three assumptions are essential for the successful implementation of MR analysis (Emdin, Khera, and Kathiresan 2017): First, there should be a significant correlation between the selected genetic variants and the risk of interest (*p* < 5 × 10^−8^); second, there must be no confounding factors that can affect the genetic variants; finally, there should be no direct association between genetic variants and the outcome. Furthermore, in order to avoid a potential reverse causation, reverse‐MR analysis was performed.

### 2.2 Data Sources

For serum galectin‐3, summary statistics data of SNPs were extracted from a large‐scale genome‐wide association study (GWAS), with a sample size of 30,931 individuals (Folkersen et al. 2020). For PD, genetic variants were derived from the International Parkinson's Disease Genomics Consortium (IPDGC), including 33,674 PD cases and 449,056 controls (Nalls et al. 2019). All of the participants in both datasets were of European ancestry. Detailed information is listed in Table 1. The original GWASs have obtained adequate patient consent and ethical approval. Therefore, no separate ethical approval was required in the study.

**TABLE 1:** Detailed information of the studies included in Mendelian randomization analysis.

Phenotype	GWAS ID	Sample size (cases/controls)	Number of SNPs	Population	Consortium	Year	Journal	References
Serum galectin‐3	ebi‐a‐GCST90012009	21,758	13,138,257	European	—	2020	*Nature Metabolism*	Folkersen et al. (2020)
Parkinson's disease	ieu‐b‐7	33,674/449,056	17,891,936	European	IPDGC	2019	*Lancet Neurology*	Nalls et al. (2019)

Abbreviation: IPDGC, International Parkinson's Disease Genomics Consortium.

### 2.3 Selection of Genetic Instruments

For eligible instrumental variables (IVs), a series of quality control processes were constructed. We selected SNPs with a genome‐wide significant *p*‐value (*p* < 5 × 10^−8^) as the potential IVs for exposure. To avoid bias, those SNPs in linkage disequilibrium (LD, *r^2^* threshold < 0.001 within a 10‐Mb window) were excluded. Then the retained SNPs that are independent of each other were extracted. In addition, if the outcome dataset did not contain the instrumental SNPs, a highly correlated SNP in LD (*r^2^ >* 0.8) was used as a proxy.

Next, we calculated F‐statistics to avoid weak instrument bias. The F‐statistic of an SNP with a value > 10 was considered to be robust enough for MR analysis (Brion, Shakhbazov, and Visscher 2013). F‐statistics were calculated for each SNP based on the accepted formula: F‐statistic = *r* ^2^ × (*N* − 2) / (1 − *r^2^*), where *r^2^* was calculated using the formula: *r^2^* = 2 × EAF × (1 − EAF) × *β^2^*, where *r^2^* represented the proportion of the exposure explained by the genetic variants, *N* was the exposure GWAS sample size, EAF was the effect allele frequency, and *β* was the estimated effect on the exposure. Furthermore, we manually searched the PhenoScanner database (Version 2, http://www.phenoscanner.medschl.cam.ac.uk/) (Staley et al. 2016) to screen the SNP that were directly associated with the potential confounders (urate levels, homocysteine levels, cardio‐cerebrovascular diseases, cigarette smoking and coffee consumption, etc.) and outcome.

### 2.4 Statistical Analysis

The primary analysis was performed using the inverse‐variance weighting (IVW) method, which provided the most convincing results (Burgess et al. 2019). Alternative methods were conducted as sensitivity analysis, including weighted median, MR‐Egger, simple mode, weighted mode, and MR‐pleiotropy residual sum and outlier (MR‐PRESSO) (Bowden et al. 2016; Hartwig, Davey, and Bowden 2017). Statistical significance was determined by a *p*‐value of 0.05. In addition, Cochran's *Q* test and leave‐one‐out analysis were used to detect heterogeneity across the SNPs (Cohen et al. 2015), while the MR‐Egger intercept test, the MR‐PRESSO global test, and visual inspection of the funnel plot were used to detect potential horizontal pleiotropy (Verbanck et al. 2018). A *p*‐value of > 0.05 suggested no evidence of heterogeneity or pleiotropy.

The statistical analyses were conducted using R software (version 4.1.1) and the packages TwoSampleMR (Hemani et al. 2018) and MR‐PRESSO (Verbanck et al. 2018).

## 3 Results

### 3.1 Selection of IVs

Eight SNPs significantly associated with serum galectin‐3 level were extracted after screening with a *p*‐value of < 5 × 10^−8^, and removing SNPs being in LD. Then, the SNPs were scanned in the PhenoScanner. None of the chosen SNPs was directly associated with the outcome (PD) or confounding traits. Besides, F‐statistics for all SNPs ranged from 34 to 1593, indicating that they were robust enough for MR analysis. Detailed information of the IVs is shown in Table S1.

### 3.2 MR Analysis

**FIGURE 2:**
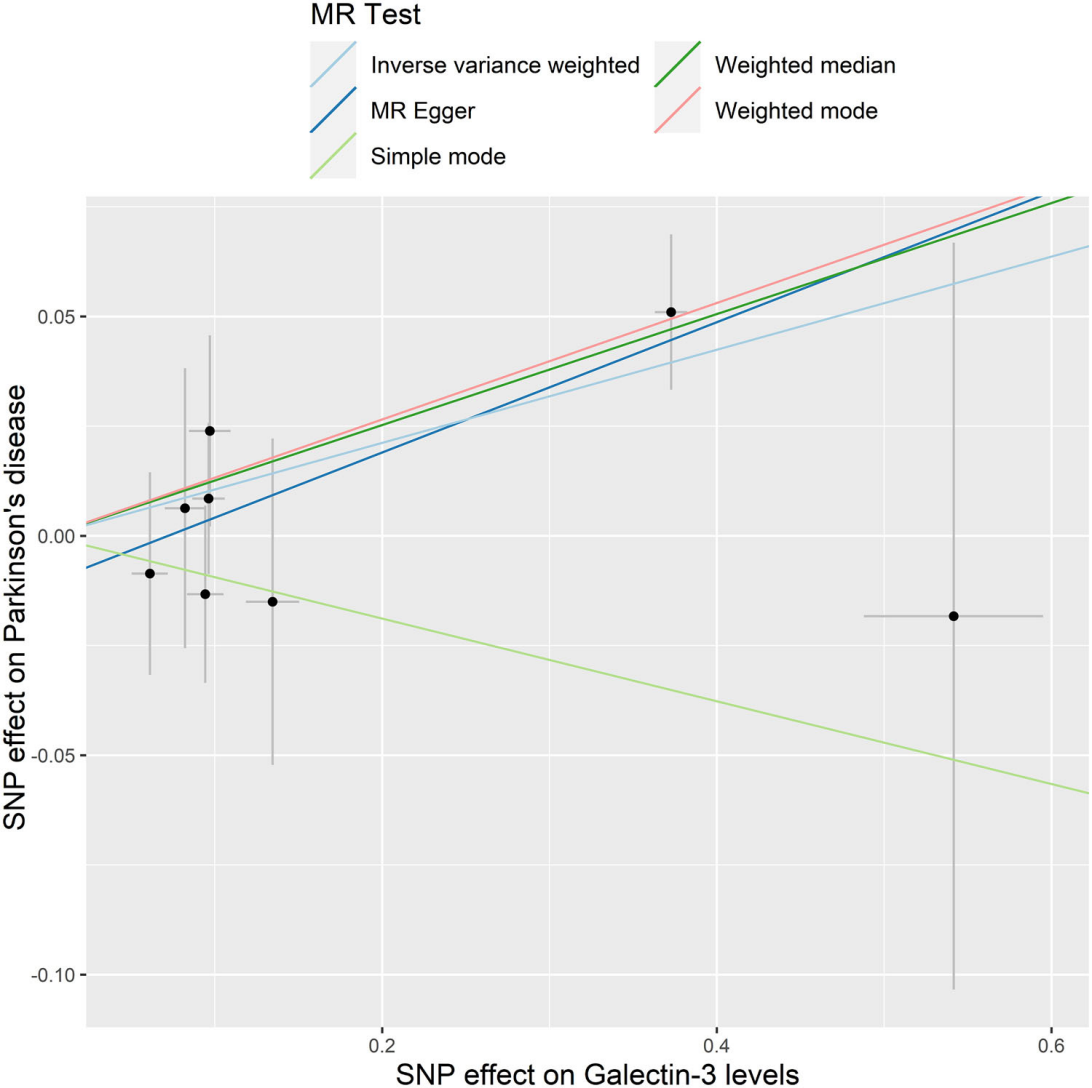
Scatter plot of the effect of serum galectin‐3 on the risk of PD. Each line represents the estimated association of different MR methods. MR, Mendelian randomization; PD, Parkinson's disease; SNP, single‐nucleotide polymorphism.

**FIGURE 3:**
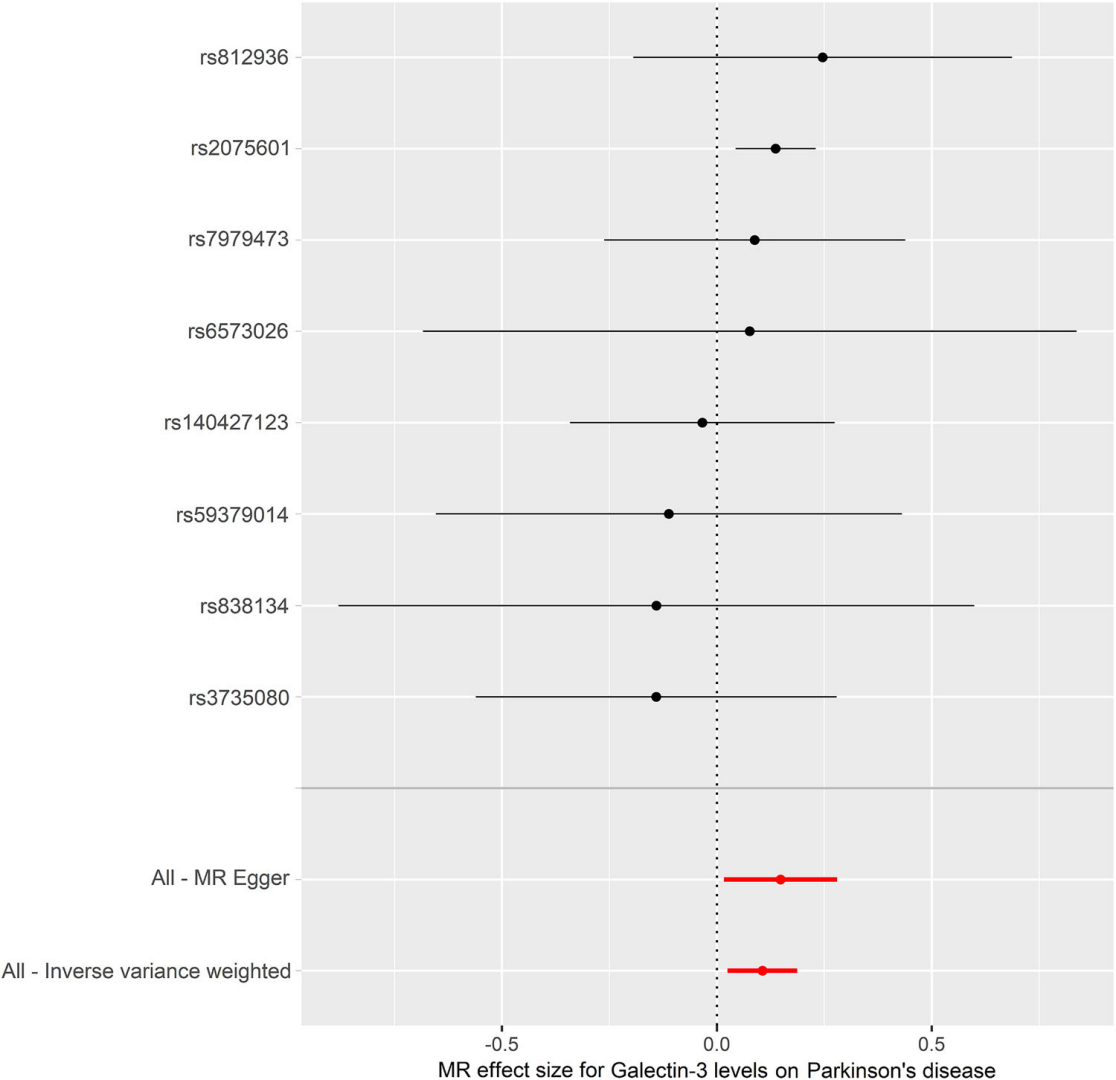
Forest plot of the effect of serum galectin‐3 on the risk of PD. Estimated betas are represented by black dots, and 95% CIs are represented by horizontal lines. CI, confidence interval; IVW, inverse variance weighting; MR, Mendelian randomization; PD, Parkinson's disease.

The IVW model suggested that a higher risk of PD was associated with each standard deviation (SD) increase in serum galectin‐3 (OR: 1.112, 95% CI: 1.025–1.206, *p* = 0.010). Similarly, MR analysis using the MR‐PRESSO (OR: 1.112, 95% CI: 1.046–1.182, *p* = 0.012), weighted median (OR: 1.135, 95% CI: 1.037–1.242, *p* = 0.006), and weighted mode (OR: 1.142, 95% CI: 1.038–1.257, *p* = 0.030) yielded consistent results (Table 2). The scatter plot and the forest plot of the effects of galectin‐3‐associated SNPs on PD are demonstrated in Figures 2 and 3.

**TABLE 2:** Association between galectin‐3 and Parkinson's disease using the Mendelian randomization analysis.

Exposure	*n*SNPs	Method	OR (95% CI)	*p‐*value
Serum galectin‐3 levels	8	IVW	1.112 (1.025 to 1.206)	**0.010**
		MR‐Egger	1.160 (1.017 to 1.323)	0.069
		MR‐PRESSO	1.112 (1.046 to 1.182)	**0.012**
		Weighted Median	1.135 (1.037 to 1.242)	**0.006**
		Weighted mode	1.142 (1.038 to 1.257)	**0.030**
		Simple mode	0.910 (0.721 to 1.148)	0.453

*Note*: Bold values indicated statistical significance (*p *< 0.05).

Abbreviations: MR, Mendelian randomization; *n*SNPs, number of single nucleotide polymorphisms; IVW, inverse‐variance weighted; MR‐PRESSO, MR‐pleiotropy residual sum and outlier; OR, odds ratio; CI, confidence interval.

**FIGURE 4:**
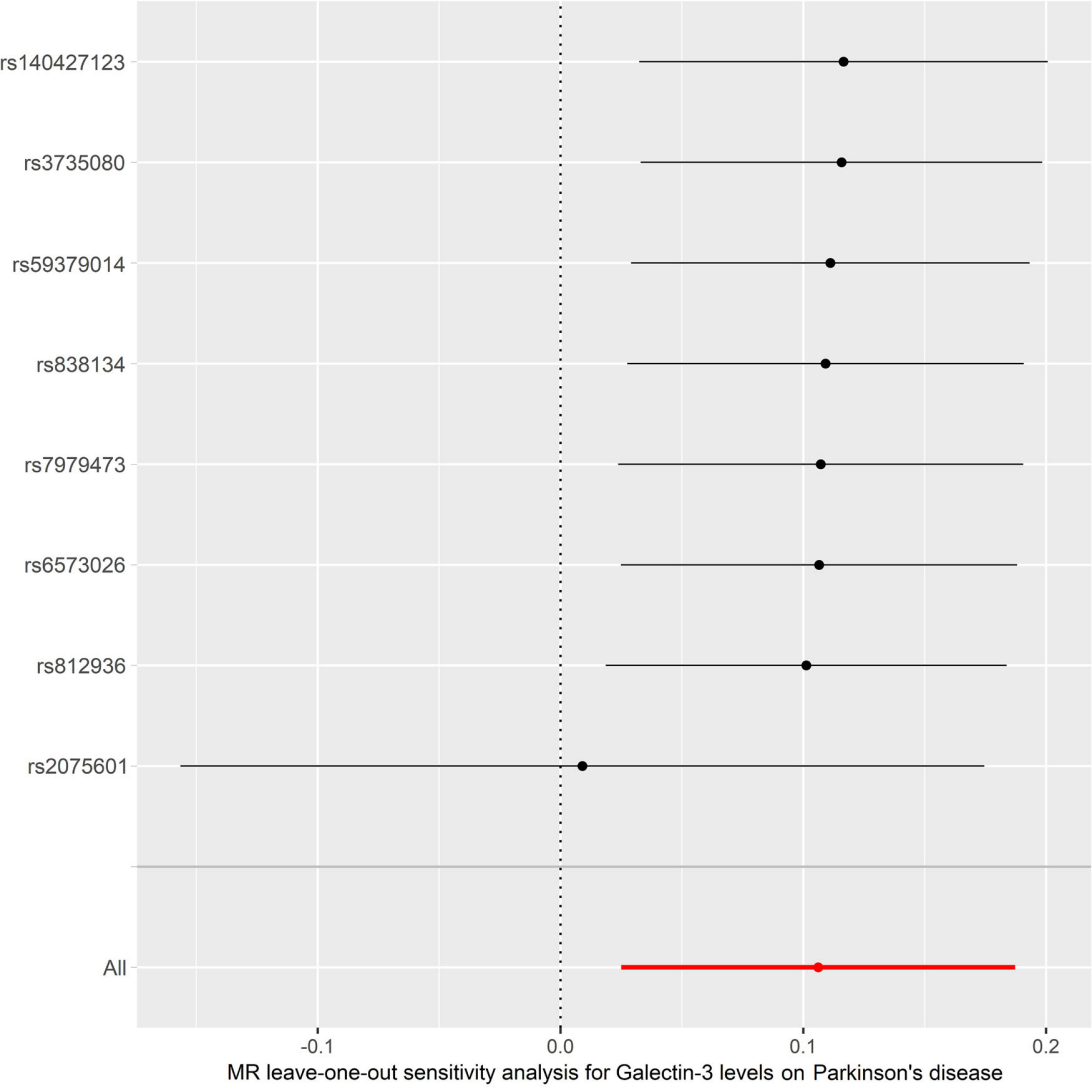
Leave‐one‐SNP‐out sensitivity analysis for serum galectin‐3 on PD. MR estimate effects were recalculated after each SNP was excluded sequentially. The results were not driven by any SNP. MR, Mendelian randomization; PD, Parkinson's disease; SNP, single‐nucleotide polymorphism.

Cochran's *Q* test and MR‐Egger intercept test showed no significant heterogeneity (*Q*‐value = 3.944, *P_Q_* = 0.780) or horizontal pleiotropy (intercept = −0.011, *p*_intercept_ = 0.454). MR‐PRESSO global test did not reveal any outliers that could generate substantial pleiotropy (*p* = 0.573) (Table 3). The funnel plot is shown in Figure S1. Besides, leave‐one‐out analysis showed that no SNP could significantly influence our MR analyses (Figure 4).

**TABLE 3:** Heterogeneity and pleiotropy tests for the associations of serum galectin‐3 levels with Parkinson's disease.

Outcome	Cochrane's *Q* test		MR‐Egger intercept test		MRPRESSO global test
	*Q*‐value	*P_Q_*	Intercept	*P*_intercept_	*p*‐value
Parkinson's disease	3.944	0.780	−0.011	0.454	0.573

Abbreviation: MR‐PRESSO, MR‐pleiotropy residual sum and outlier.

### 3.3 Reverse‐MR Analysis

A reverse‐MR analysis was performed to examine the possibility of reverse causality. A total of 22 SNPs were chosen from PD GWAS summary statistics (see Table S2). None of the methods, including IVW, MR‐Egger, MR‐PRESSO, weighted median, weighted mode, and simple mode, could reveal a causal effect of PD on serum galectin‐3 level (see Table S3). The scatter plot, forest plot, leave‐one‐out, and funnel plot are presented in Figures S2–S5.

## 4 Discussion

The study evaluated the relationship between genetically predicted serum galectin‐3 and PD using a bidirectional MR approach. Our findings provide further evidence for the suggestive association between serum galectin‐3 and PD.

Galectin‐3 is a *β*‐galactoside‐binding lectin, which is expressed widely in various tissues and organs (Srejovic et al. 2020; Papaspyridonos et al. 2008). As a cytosolic protein, galectin‐3 is synthesized by free ribosomes (Li et al. 2020; Wang and Guo 2016). Studies have indicated that it is involved in numerous activities, including cell adhesion, migration, proliferation, apoptosis, and regulation of inflammatory responses (Dong et al. 2018). The association between galectin‐3 and PD is raised by the discovery of increased galectin‐3 in the serum of the PD patients (Yazar, Yazar, and Cihan 2019). Receiver operating characteristics curve analysis based on clinical data has indicated that the best predictability of PD was at a serum galectin‐3 level of 1720.06 pg/mL, with a sensitivity of 68.33% and a specificity of 93.33% (Cengiz et al. 2019). Additionally, increased level of galectin‐3 was involved in PD progression (Wu et al. 2021). These observational findings seemed to be consistent with our results, that an increase of one SD in the genetically determined serum galectin‐3 level corresponded to an 11.2% increase in the risk of PD, suggesting a detrimental effect of increased galectin‐3 on the disease. In recent years, researchers have recognized that gut–brain interaction plays an important role in PD. Considerable evidence has proved that PD could be possibly originated from peripheral inflammation, especially gut inflammation (Perez‐Pardo et al. 2017; Kim et al. 2019; Anis et al. 2022; Kelly et al. 2016). Specifically, galectin‐3 is reported to be deeply involved in gut inflammation. It can identify *β*‐galectocide molecules expressed by bacteria in the gut and promote the recruitment of macrophages and neutrophils to the site of infection (Boziki et al. 2018; Fowler et al. 2006). The inflammation and microbial dysbiosis induced by galectin‐3 may increase the expression of peripheral *α*‐synuclein (Gorecki et al. 2019), which gradually propagates from gut to brain, inducing Parkinson's pathology (Braak et al. 2003). Furthermore, Marina et al. speculated that increased expression of peripheral galectin‐3 after *Helicobacter pylori* infection might damage the blood–brain barrier and facilitate the uptake of circulating *α*‐synuclein into the brain (Sui et al. 2014). Therefore, galectin‐3 may play a role in PD through its involvement in *α*‐synuclein pathology.

In addition, the link between galectin‐3 and PD pathology is further emphasized by increasing evidence. Galectin‐3 can be found within and surrounding LBs in post‐mortem brain sections of PD patients (Burbidge et al. 2022; Flavin et al. 2017; Garcia‐Revilla et al. 2023). Phagocytosis of *α*‐synuclein fibrils could selectively induce overexpression of galectin‐3 in vitro and in vivo (Garcia‐Revilla et al. 2022). Subsequently, increased galectin‐3 could activate microglia via toll‐like receptor pathways (Ge et al. 2022) and promote assembly of NLRP3 inflammasome (Siew et al. 2019), leading to sustained neuroinflammatory process in the brain. Besides, Juan et al. suggested that exogenous galectin‐3 could affect the stability of endogenous *α*‐synuclein fibrils and transform the preformed long fibrils into small, toxic fibrils (Garcia‐Revilla et al. 2023), which has recently been shown to be the major toxic species in PD progression (Emin et al. 2022). In vitro studies described the recruitment of galectin‐3 to damaged lysosomes bearing *α*‐synuclein fibrils in neuronal cells (Dilsizoglu et al. 2021; Jiang et al. 2017), resulting in autophagy induction of broken lysosomes (Flavin et al. 2017). These studies echo with our MR findings of suggestive involvement of galectin‐3 in the risk of PD and raise the possibility that galectin‐3 may promote the escape of *α*‐synuclein fibrils from damaged lysosomes and contribute to the seeding of *α*‐synuclein in the brain.

A notable advantage of the study is that large‐scale GWAS summary statistics were used for analysis. And the comprehensive MR design ensures the robustness and stability of our findings. Inevitably, some limitations should be considered. First, due to the restricted awareness of individual data and adjusting factors, the study failed to carry out the assessment of the associations between individual genetic variants and confounding factors comprehensively. For instance, the genetic variants associated with serum galectin‐3 may be less than ideal if the activities of the molecule could be affected by other related factors such as aging and occupational exposures. Further in vitro and in vivo studies would be needed to validate the findings. Second, the utilized GWASs data were obtained mainly from the European population, which limits the generalizability of our results to other racial/ethnic populations. Third, since the existence of the blood–brain barrier, galectin‐3 in serum is not able to reflect its changes in the brain directly.

## 5 Conclusions

In conclusion, the present study demonstrates that genetic predisposition to serum galectin‐3 is associated with the risk of PD. This provides new insight into the role of galectin‐3 in PD. Future studies are needed to clarify the exact mechanism behind our findings.

## Author Contributions

**Rui Pan**: conceptualization, methodology, writing–review and editing, formal analysis, funding acquisition. **Wei Li**: conceptualization, writing–original draft, writing–review and editing, methodology. **Jinyuan Wang**: formal analysis, writing–review and editing, visualization. **Jiarong Xie**: formal analysis, writing–review and editing. **Xiucan Weng**: writing–original draft, writing–review and editing. **Ying Yang**: writing–original draft, writing–review and editing. **Xiaolei Shi**: conceptualization, supervision, funding acquisition, methodology, writing–review and editing.

## Ethics Statement

The ethical approval for all studies employed in the analysis has been granted by the pertinent institutional review boards.

## Conflicts of Interest

The authors declare no conflicts of interest.

### Peer Review

The peer review history for this article is available at https://publons.com/publon/10.1002/brb3.70103.

## Supporting information




**Table S1**. Characteristics of selected SNPs for serum galectin‐3.
**Table S2**. Characteristics of selected SNPs for Parkinson's disease.
**Table S3**. Reverse causal relationship of serum galectin‐3 with Parkinson's disease.
**Figure S1**. funnel plot of the association between serum galectin‐3 and Parkinson's disease.
**Figure S2**. Scatter plot of the reverse causal relationship of serum galectin‐3 with Parkinson's disease.
**Figure S3**. Forest plot of the reverse causal relationship of serum galectin‐3 with Parkinson's disease.
**Figure S4**. Leave‐one‐SNP‐out sensitivity analysis for the reverse‐MR analysis.
**Figure S5**. funnel plot of the association between Parkinson's disease and serum galectin‐3, reverse MR analysis.

## Data Availability

The present MR study employed public summary‐level data of GWAS. The analyses utilized data sourced from the publicly accessible IEU Open GWAS database (https://gwas.mrcieu.ac.uk/), accessed on January 7, 2024.
