# Supplementary material for: Association Between Serum Galectin‐3 and Parkinson's Disease: A Two‐Sample Mendelian Randomization Study
Source: Brain Behav. 2024 Oct 23;14(10):e70103. doi: 10.1002/brb3.70103 (PMC11499214; doi:10.1002/brb3.70103)
Supplement: Supplementary file 1 — Table S1. Characteristics of selected SNPs for serum galectin‐3. Table S2. Characteristics of selected SNPs for Parkinson's disease. Table S3. Reverse causal relationship of serum galectin‐3 with Parkinson's disease. Figure S1. funnel plot of the association between serum galectin‐3 and Parkinson's disease. Figure S2. Scatter plot of the reverse causal relationship of serum galectin‐3 with Parkinson's disease. Figure S3. Forest plot of the reverse causal relationship of serum galectin‐3 with Parkinson's disease. Figure S4. Leave‐one‐SNP‐out sensitivity analysis for the reverse‐MR analysis. Figure S5. funnel plot of the association between Parkinson's disease and serum galectin‐3, reverse MR analysis. [file BRB3-14-e70103-s001.docx]

**Supplementary file**

**Supplementary Tables**

**Table S1.** Characteristics of selected SNPs for serum galectin-3

**Table S2.** Characteristics of selected SNPs for Parkinson’s disease

**Table S3.** Reverse causal relationship of serum galectin-3 with Parkinson’s disease

**Supplementary Figures**

**Figure S1.** funnel plot of the association between serum galectin-3 and Parkinson’s disease.

**Figure S2.** Scatter plot of the reverse causal relationship of serum galectin-3 with Parkinson’s disease.

**Figure S3.** Forest plot of the reverse causal relationship of serum galectin-3 with Parkinson’s disease.

**Figure S4.** Leave-one-SNP-out sensitivity analysis for the reverse-MR analysis.

**Figure S5.** funnel plot of the association between Parkinson’s disease and serum galectin-3, reverse MR analysis.

**Supplementary Tables**

**Table S1.** Characteristics of selected SNPs for serum galectin-3

| **SNP** | **Trait** | **Chr** | **Pos.** | **Effect allele** | **Other allele** | **EAF** | **Beta** | **SE** | ***P value*** | **R^2^** | **F statistic** |
| --- | --- | --- | --- | --- | --- | --- | --- | --- | --- | --- | --- |
| rs3735080 | Galectin-3 | 7 | 150217309 | T | C | 0.2395 | 0.0943 | 0.0109 | 3.97832E-18 | 0.003239 | 70.696671 |
| rs59379014 | Galectin-3 | 11 | 126228000 | T | C | 0.0771 | 0.1345 | 0.0159 | 2.64119E-17 | 0.002574 | 56.14446 |
| rs7979473 | Galectin-3 | 12 | 121420260 | G | A | 0.6025 | -0.0963 | 0.0097 | 3.71279E-23 | 0.004442 | 97.071343 |
| rs140427123 | Galectin-3 | 14 | 55526381 | G | A | 0.0128 | -0.5415 | 0.0536 | 5.50047E-24 | 0.00741 | 162.415459 |
| rs6573026 | Galectin-3 | 14 | 55892017 | T | C | 0.7859 | -0.0822 | 0.012 | 6.97268E-12 | 0.002274 | 49.585902 |
| rs2075601 | Galectin-3 | 14 | 55609234 | T | C | 0.433 | -0.3727 | 0.0097 | 1.00E-200 | 0.068206 | 1592.508361 |
| rs812936 | Galectin-3 | 19 | 5844649 | A | G | 0.8036 | 0.097 | 0.0124 | 6.27914E-15 | 0.00297 | 64.807799 |
| rs838134 | Galectin-3 | 19 | 49257739 | C | A | 0.292 | -0.0612 | 0.0108 | 1.217E-08 | 0.001549 | 33.752326 |

Chr, chromosome; EAF, Effect allele frequency; Pos, position; SE, standard error; SNP, single-nucleotide polymorphism. The threshold was set at *P* < 1×10^-5^.

**Table S2.** Characteristics of selected SNPs for Parkinson’s disease

| **SNP** | **Trait** | **Chr** | **Pos.** | **Effect allele** | **Other allele** | **EAF** | **Beta** | **SE** | ***P value*** | **R^2^** | **F statistic** |
| --- | --- | --- | --- | --- | --- | --- | --- | --- | --- | --- | --- |
| rs35749011 | PD | 1 | 155135036 | A | G | 0.0191 | 0.7508 | 0.0659 | 5.02227E-30 | 0.021122 | 10416.191615 |
| rs823106 | PD | 1 | 205656453 | C | G | 0.8488 | -0.1492 | 0.0239 | 4.09996E-10 | 0.005714 | 2774.159338 |
| rs4613239 | PD | 2 | 169119609 | G | C | 0.1326 | 0.1784 | 0.0248 | 6.21441E-13 | 0.007321 | 3560.115292 |
| rs6741007 | PD | 2 | 135537119 | G | T | 0.4507 | -0.1233 | 0.0175 | 2.08689E-12 | 0.007528 | 3661.540461 |
| rs4488803 | PD | 3 | 58218352 | A | G | 0.3746 | -0.1136 | 0.0199 | 1.07599E-08 | 0.006047 | 2936.815137 |
| rs10513789 | PD | 3 | 182760073 | G | T | 0.1826 | -0.1596 | 0.0219 | 3.18493E-13 | 0.007604 | 3698.789306 |
| rs7695720 | PD | 4 | 77183300 | C | A | 0.2091 | -0.1255 | 0.0208 | 1.52799E-09 | 0.005209 | 2527.696925 |
| rs34311866 | PD | 4 | 951947 | C | T | 0.1958 | 0.2272 | 0.0231 | 7.97444E-23 | 0.016256 | 7977.898835 |
| rs4698412 | PD | 4 | 15737348 | A | G | 0.553 | 0.1258 | 0.0168 | 7.04855E-14 | 0.007824 | 3806.647079 |
| rs356203 | PD | 4 | 90666041 | T | C | 0.6169 | -0.2398 | 0.0178 | 3.00677E-41 | 0.027180 | 13487.127156 |
| rs75646569 | PD | 5 | 60345424 | G | T | 0.1117 | 0.1916 | 0.0266 | 5.61824E-13 | 0.007285 | 3542.480450 |
| rs35265698 | PD | 6 | 32561334 | G | C | 0.1547 | -0.2000 | 0.0303 | 3.92735E-11 | 0.010461 | 5103.202206 |
| rs858295 | PD | 7 | 23245569 | G | A | 0.3947 | -0.1039 | 0.0176 | 3.83098E-09 | 0.005158 | 2502.820573 |
| rs620490 | PD | 8 | 16697579 | G | T | 0.2762 | -0.1174 | 0.019 | 6.45595E-10 | 0.005511 | 2674.056243 |
| rs144814361 | PD | 10 | 121410917 | T | C | 0.0174 | 0.4411 | 0.068 | 9.06564E-11 | 0.006653 | 3233.099193 |
| rs329647 | PD | 11 | 133764666 | C | G | 0.6662 | -0.1133 | 0.0178 | 1.94402E-10 | 0.005709 | 2771.717889 |
| rs75505347 | PD | 12 | 40885549 | T | C | 0.0195 | 0.3917 | 0.0674 | 6.11702E-09 | 0.005867 | 2848.879552 |
| rs10847864 | PD | 12 | 123326598 | T | G | 0.3625 | 0.1274 | 0.0179 | 9.81296E-13 | 0.007502 | 3648.798744 |
| rs4774417 | PD | 15 | 61993702 | A | G | 0.7397 | 0.1052 | 0.0192 | 4.62605E-08 | 0.004262 | 2066.192850 |
| rs12934900 | PD | 16 | 30923602 | T | A | 0.6571 | 0.1215 | 0.0184 | 4.33112E-11 | 0.006652 | 3232.609978 |
| rs58879558 | PD | 17 | 44095467 | C | T | 0.2229 | -0.2383 | 0.025 | 1.36301E-21 | 0.019673 | 9687.285920 |
| rs4588066 | PD | 18 | 40672964 | A | G | 0.326 | 0.1046 | 0.0178 | 4.45297E-09 | 0.004808 | 2332.169294 |

PD, Parkinson’s disease; Chr, chromosome; EAF, Effect allele frequency; Pos, position; SE, standard error; SNP, single-nucleotide polymorphism. The threshold was set at *P* < 1×10^-5^.

**Table S3.** Reverse causal relationship of serum galectin-3 with Parkinson’s disease

| **Exposure** | **nSNPs** | **Method** | **OR (95% CI)** | ***P* value** | **Q pval** | **intercept**  ***p* value** | **Global *P*** |
| --- | --- | --- | --- | --- | --- | --- | --- |
| Parkinson’s disease | 22 | IVW | 1.031 (0.991 to 1.072) | 0.131 | 0.030 |  |  |
|  |  | MR-Egger | 1.036 (0.932 to 1.151) | 0.518 |  | 0.917 |  |
|  |  | MR-PRESSO | 1.031 (0.993 to 1.071) | 0.128 |  |  | 0.059 |
|  |  | Weighted Median | 1.022 (0.978 to 1.069) | 0.331 |  |  |  |
|  |  | Weighted mode | 1.022 (0.967 to 1.079) | 0.457 |  |  |  |
|  |  | Simple mode | 1.022 (0.967 to 1.079) | 0.537 |  |  |  |

MR, Mendelian randomization; nSNPs, number of single nucleotide polymorphisms; IVW, inverse-variance weighted; MR-PRESSO, Pleiotropy Residual Sum and Outlier; OR, odds ratio; CI, confdence interval; Q_pval, *P*-value of the Cochran Q statistic.

**Supplementary Figures**

**
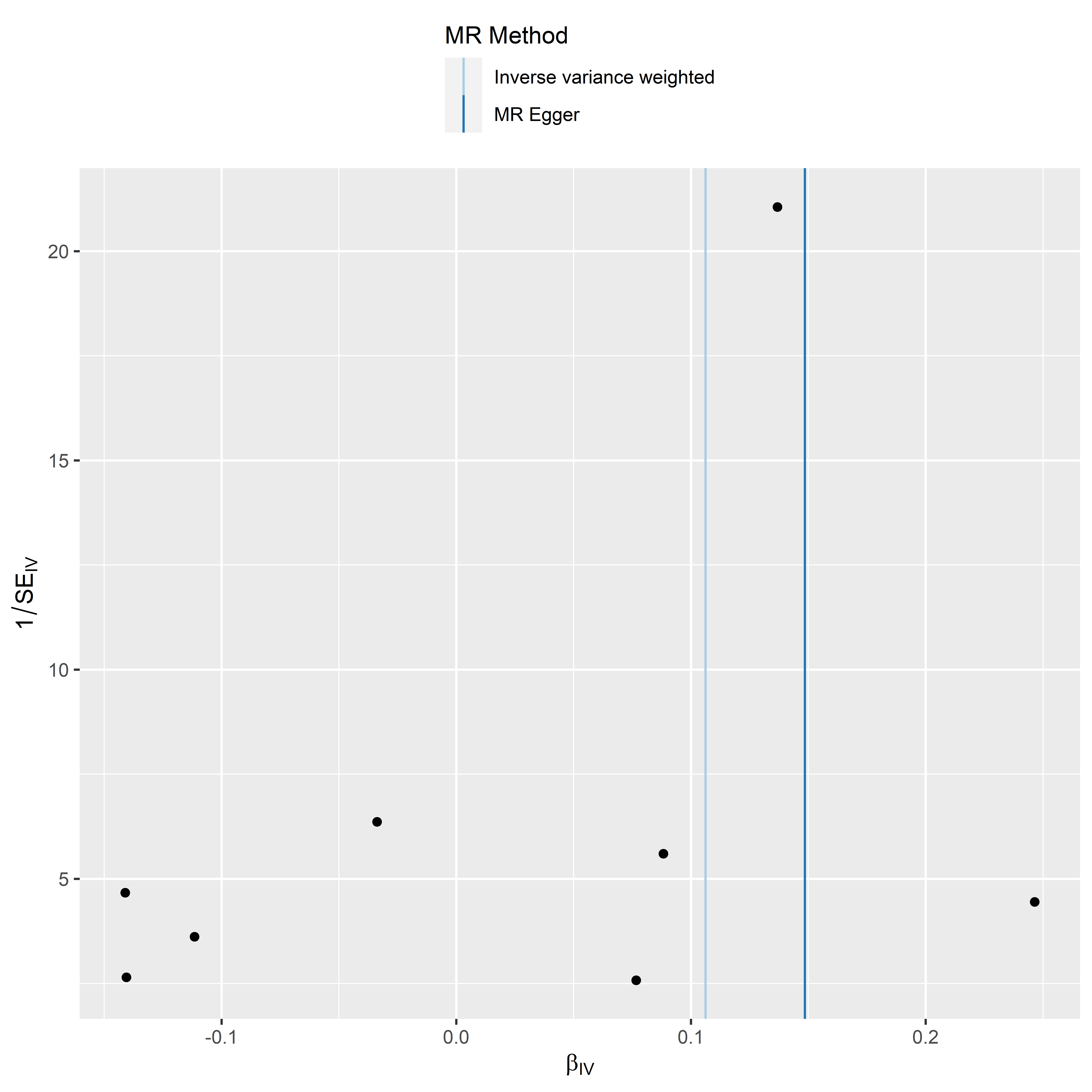
**

**Figure S1.** funnel plot of the association between serum galectin-3 and PD.

PD, Parkinson’s disease.


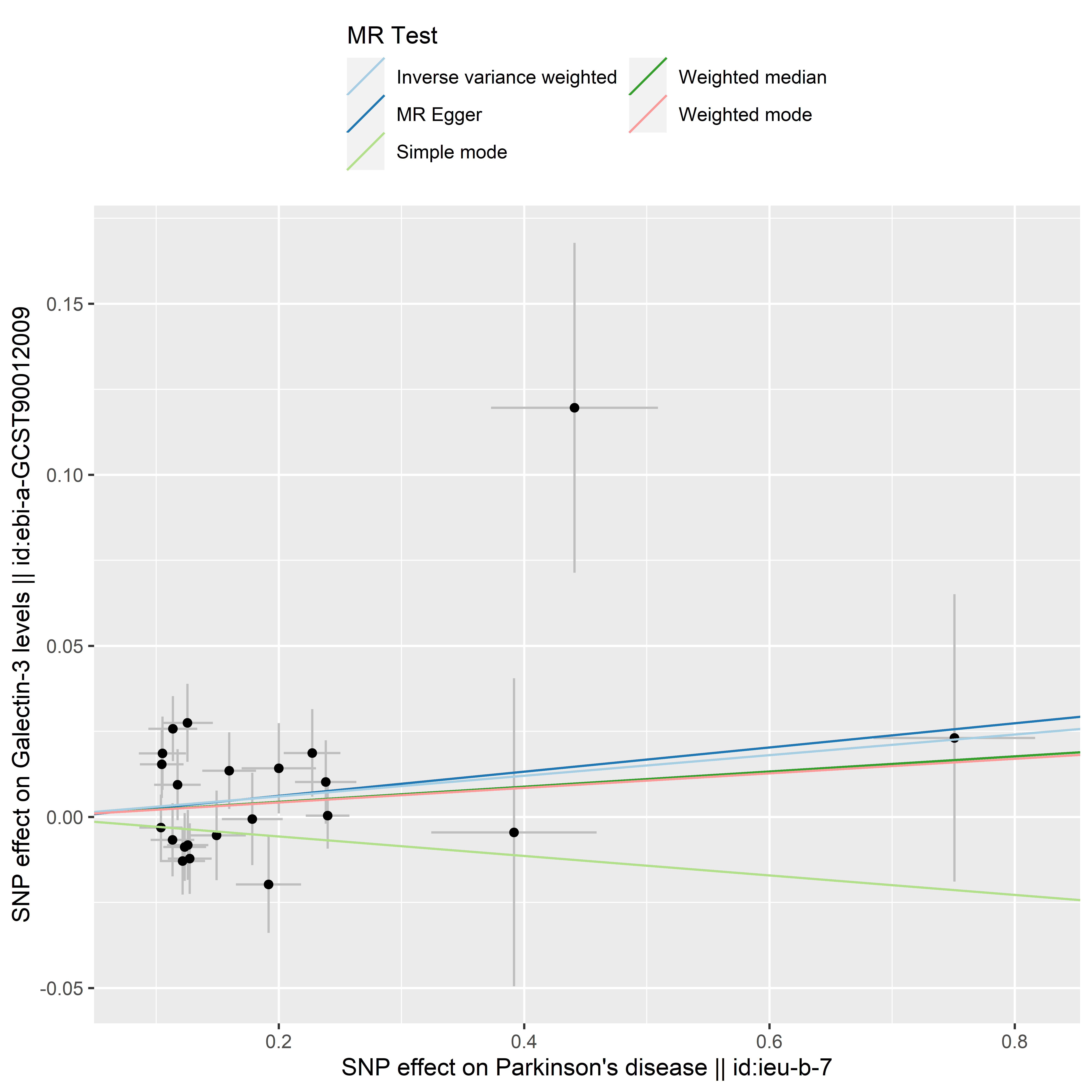


**Figure S2.** Scatter plot of the reverse causal relationship of serum galectin-3 with PD.

PD, Parkinson’s disease.


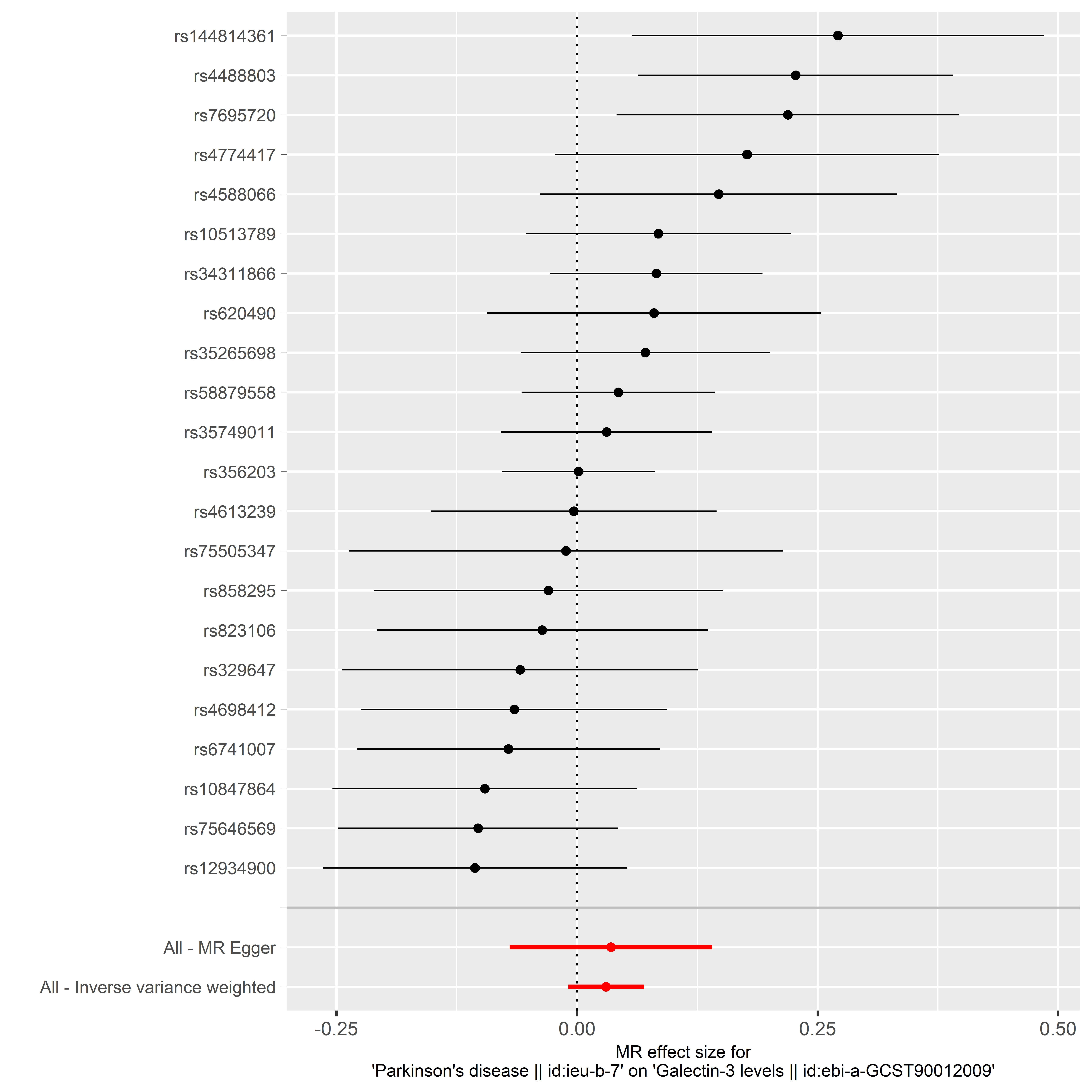


**Figure S3.** Forest plot of the reverse causal relationship of serum galectin-3 with PD.

PD, Parkinson’s disease.


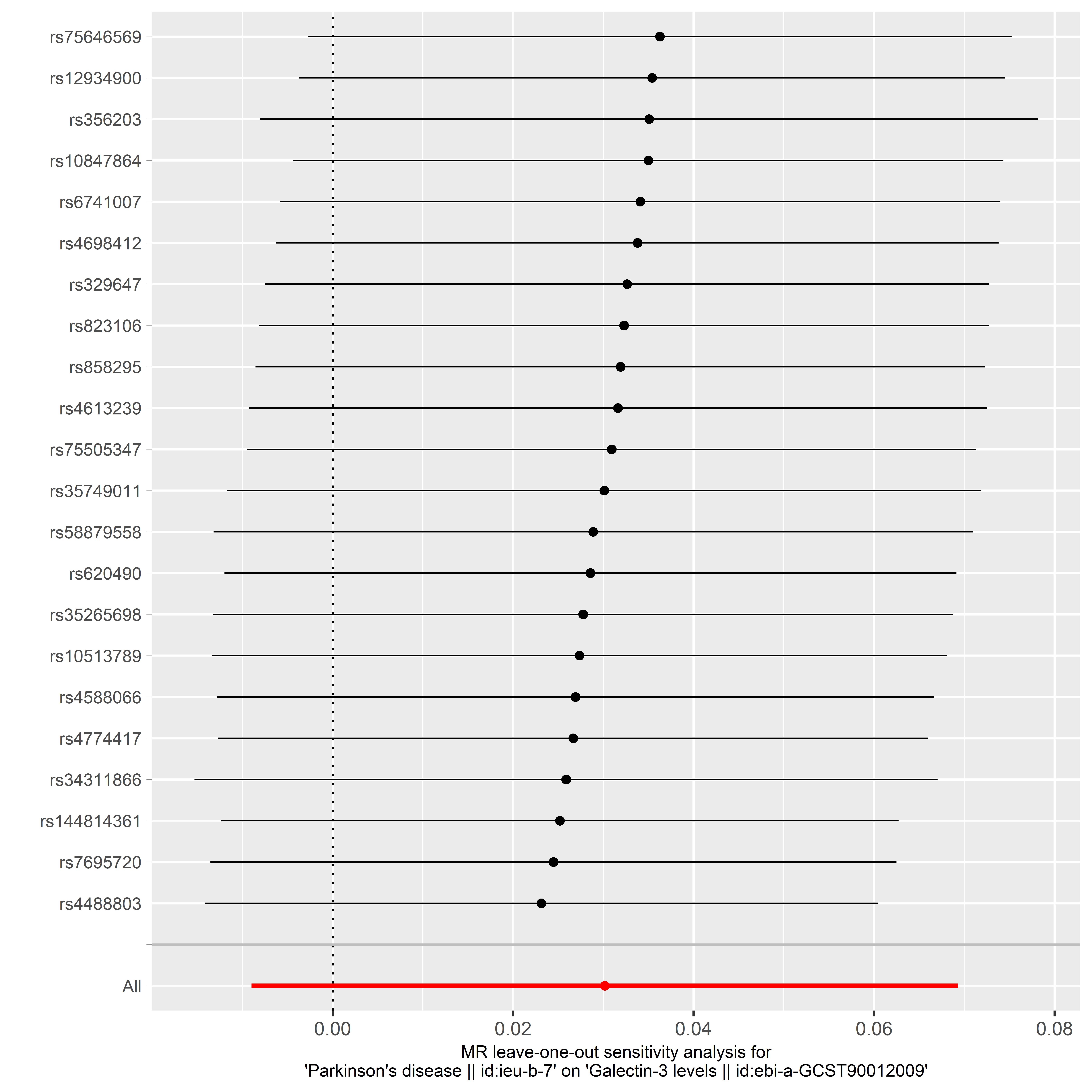


**Figure S4.** Leave-one-SNP-out sensitivity analysis for the reverse-MR analysis.


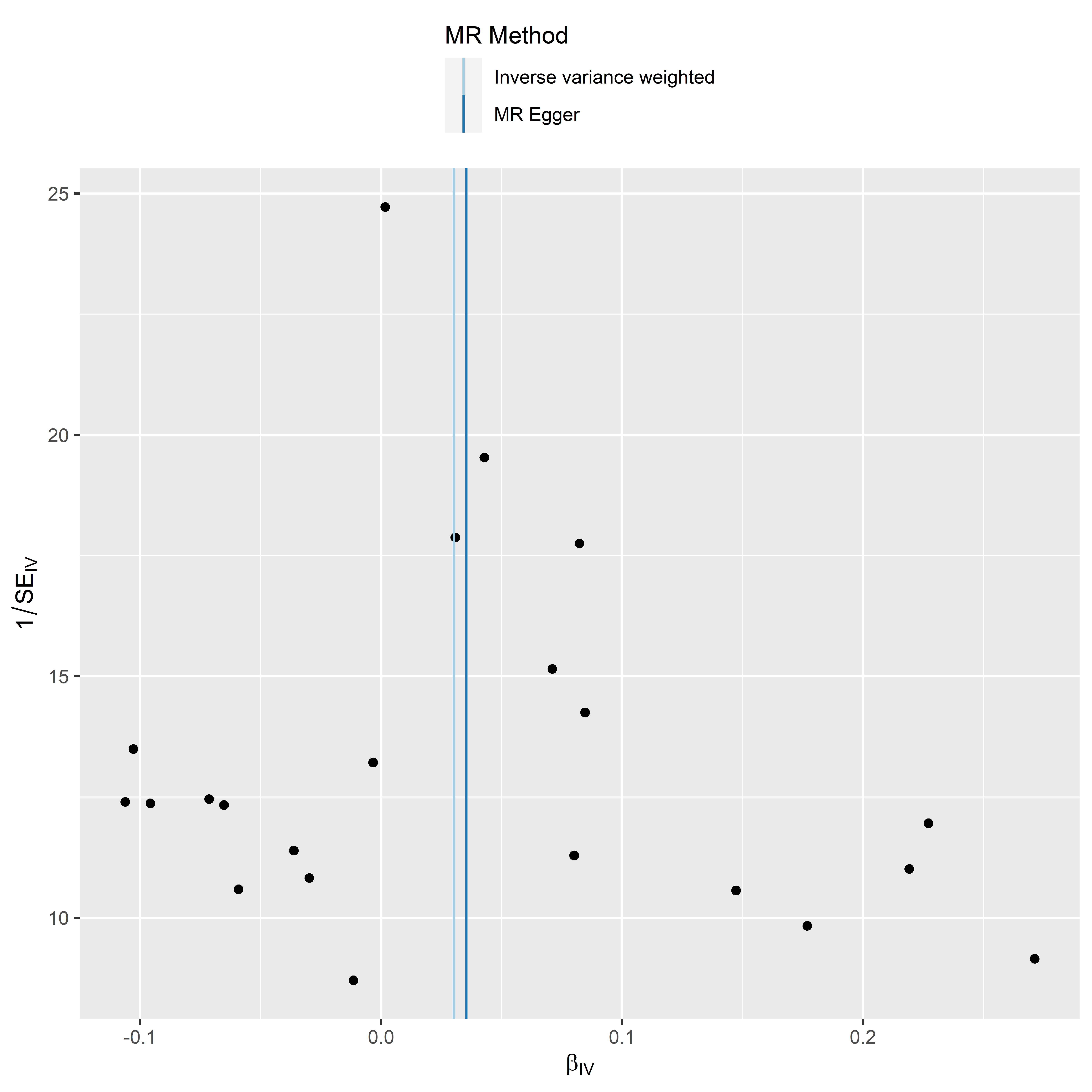


**Figure S5.** funnel plot of the association between PD and serum galectin-3, reverse MR analysis.

PD, Parkinson’s disease.
